# Terbinafine-induced Toxic Epidermal Necrolysis in Pregnancy: A Case Report

**DOI:** 10.31729/jnma.8116

**Published:** 2023-04-30

**Authors:** Ramesh Lamichhane, Mahendra Raj Pandey, Ayushma Dhungana, Anjana Lamsal

**Affiliations:** 1 Department of Internal Medicine, Nepal Cardio Diabetes and Thyroid Center, Budhanilkantha, Kathmandu, Nepal; 2 Department of Dermatology, National Medical College and Teaching Hospital, Bhediyahi, Birgunj, Nepal

**Keywords:** *case reports*, *pregnancy*, *Stevens-Johnson syndrome*, *toxic epidermal necrolysis*

## Abstract

Toxic epidermal necrolysis in pregnancy is a rare disease that can have an adverse effect on pregnancy outcomes. Common etiology of the condition includes medication triggered followed by mycoplasma infection. Almost one-third of cases are idiopathic. Despite the rarity of data, terbinafine causing toxic epidermal necrolysis has been reported. Toxic epidermal necrolysis manifests as a macule, erythema followed by a blister in the chest and spreading to other parts of the body. Removal of the offending agent and supportive management is the cornerstone of management. Here we report 22-year-old primipara pregnant women presenting with toxic epidermal necrolysis after 3 weeks of oral terbinafine use with good pregnancy outcomes.

## INTRODUCTION

Steven johnson syndrome (SJS) and toxic epidermal necrolysis (TEN) are rare immune-mediated mucocutaneous reactions characterized by necrosis and epidermal detachment. The annual incidence is approximately 2 to 7 per million population where SJS is more common than TEN.^1^ Data regarding its incidence in pregnancy is limited. Among medications, terbinafine has been described as a potential trigger.^2^ Here, we report a case of a 22-year-old primipara patient at 28 weeks of gestation pregnant women who developed TEN after the use of oral terbinafine for dermatophyte infection.

## CASE REPORT

A 22-year-old primipara patient at 28 weeks of gestation was admitted to Department of dermatology with complaints of swelling of the face and simultaneous blisters over the chest, neck, and legs for 5 days. One month ago, she had patchy hair loss and scaling for which she was prescribed oral terbinafine in a rural setting with suspicion of dermatophyte infection. Her symptoms got worsened after these medications. She started having erythematous macules, and atypical targetoid lesions with blisters starting at the chest and later involving other body areas. The blister was preceded by erythema. The symptoms of facial swelling and blister got worse and then she was referred to our hospital.

**Figure 1:**
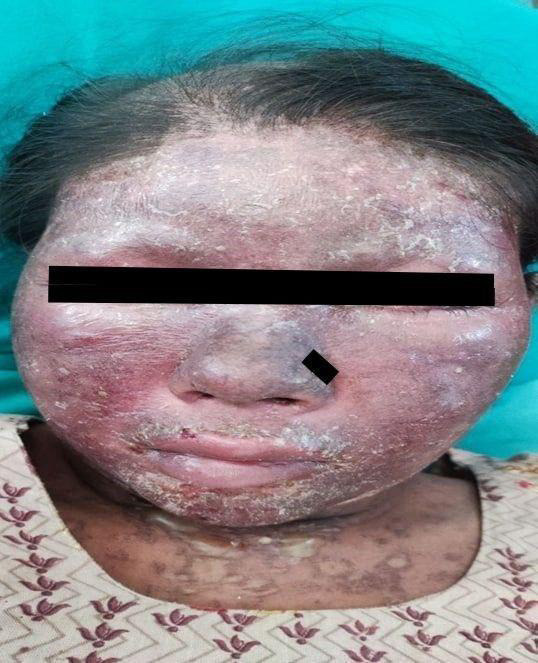
Facial involvement at the time of presentation.

The patient's general condition was poor. Her BP was 110/70 mm Hg, pulse 96 bpm, and temperature 98.6^o^ F. In addition, bipedal edema was present. On mucocutaneous examination, there were patches of hair loss with an erythematous base on the scalp. Moreover, the face was swollen including the eyelids and lips (Figure 1).

**Figure 2:**
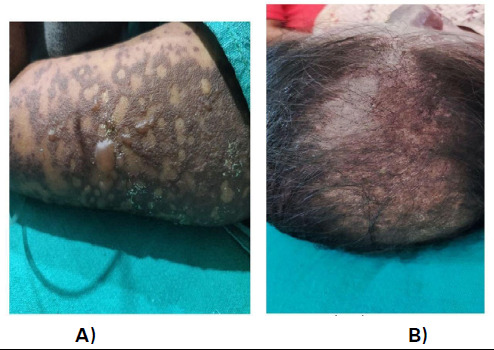
A) Bullae in the left arm, B) Involvement of scalp.

Erosion was also seen in the buccal mucosa over the soft and hard palate. A widespread erythematous rash with vesicles and bulla was observed in the scalp, chest, and upper extremities (Figure 2).

Nikolsky's sign was also positive. Obstetric examination showed a fundal height of 28 weeks, longitudinal lie with normal fetal heart sound. Cardiovascular and neurological examinations were normal.

The hematological investigations revealed hemoglobin of 9.6 gm/dl, bicarbonate 18.8 mmol/l, and a normal leukocyte count. Liver enzymes, renal function tests, blood glucose, serum albumin, and coagulation profile were all within normal limits. Ultrasound (USG) of pregnancy profile revealed normal appropriate for gestational age fetus with cephalic presentation.

Based on the history of drug intake followed by a cascade of dermatologic manifestations involving 36% of body surface area (BSA), a diagnosis of TEN was made. The BSA is calculated using the Modified Lund-Browder chart. The patient had involvement head (9%), both arms (18%), and anterior trunk (18%) resulting in a total surface area of 36%. The prognosis of this condition was calculated using the SCORTEN prognosis scoring system which revealed a score of 2 (BSA involved on day 1 >10% and serum bicarbonate level <20 mmol). This prognosis score corresponds to the mortality rate of 12.1%.

Initial management involved immediate discontinuation of terbinafine, supportive care, and adjunctive corticosteroid therapy. Intravenous (IV) dexamethasone 16 mg on the first day which was tapered by 2 mg on each consecutive couple of days. In addition, a moderately potent topical steroid was also used. Hydration and nutritional status were maintained with intravenous fluids along with a high-protein diet. Wound care was done with daily potassium permanganate dressing.

**Figure 3:**
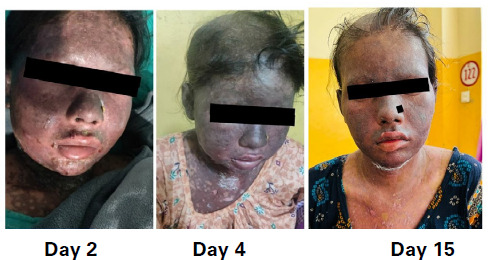
Showing hospitalization A) Day 2, B) Day 4, and C) Day 15.

Over the period of 2 weeks, the patient's blister started healing and her general condition got improved (Figure 3).

Although there were residual hypopigmented and hyperpigmented in the healing area. Periodic obstetric examinations revealed no complications in the fetus and pregnancy itself. The patient was discharged on the 15^th^ day of admission. As the patient lived far from our facility, the patient was counseled about future drug avoidance, implication, and regular follow-up in a nearby medical center. The patient came to our hospital for obstetric care where she delivered a 2.3 kg healthy baby without any complications.

## DISCUSSION

SJS and TEN indicate the same disease but are differentiated based on the severity of skin involvement. Less than 10% body surface area (BSA) involvement is termed as SJS, more than 30% BSA involvement is termed as TEN, and 10-30% BSA is called SJS/TEN overlap. The pathogenesis of this condition is not well understood but it is believed to be a type IV hypersensitivity reaction that stimulates cytotoxic T cells and releases granulysin.^3^ The risk of disease increases in patients with malignancy, and underlying immunologic diseases.^4^

Medications followed by mycoplasma pneumonia are the most common trigger for TEN. In more than one-third of the patient's cause is unknown.^5^ Among drugs allopurinol, anticonvulsants (lamotrigine, phenytoin, carbamazepine), antibiotics (sulfa drugs), sulfasalazine, nevirapine, and oxicam NSAIDs are most commonly implicated.^6^ Terbinafine has been suspected as a trigger and reported.^2^ In our case terbinafine triggered the disease.

The clinical course consists of the prodromal phase followed by mucocutaneous lesions. Influenza-like symptoms precede 1-3 days before involving skin and mucus membranes. Mucous membranes are almost always involved and may precede to development of cutaneous manifestation.^7^ The prodromal phase usually begins 1-3 weeks after taking medication. In our case patient's symptoms started 3 weeks later. Lesions usually start in the chest followed by spreading into other areas. The scalp is typically spared but was involved in our case. Ocular involvement is seen in 80% of cases. The eye involvement ranges from conjunctival hyperemia to surface epithelial defect and pseudomembrane formation. Our patient had conjunctival erythema only indicating mild ocular involvement.

Diagnosis of SJS/TEN is primarily clinical. Histopathological findings are supportive but not specific. Keratinocyte necrosis is a hallmark finding. Hematologic manifestations include anemia and lymphopenia.^8^ In our case anemia was present. Blood urea nitrogen and blood glucose are considered severity markers, which was normal in our case.^9^

Patients with suspected SJS/TEN should be evaluated in the hospital. Severity and prognosis should be determined after diagnosis. The prognosis is calculated by SCORTEN score, which was 2, indicating a mortality rate of 12.1%. Patients with involvement of ≥10 percent require treatment in the ICU/burn center/ specialized dermatology unit. Prompt identification and discontinuation of offending medication should be done immediately. In our case, the temporal sequence of symptoms after using terbinafine indicates causality.

Supportive therapy is the mainstay of management which involves fluid, nutrition, pain, and wound management. Infection leading to bacteremia and sepsis is the leading cause of death. Adjunctive pharmacotherapies like cyclosporine, systemic glucocorticoids, anti-tumor necrosis factor, and intravenous immune globulin (IVIG) have been used but data regarding the efficacy of its use are limited. However, only a few studies have shown reduced mortality with the use of corticosteroids we used corticosteroids and supportive management.^10^ Recurrence with the use of the same or similar group of drugs can occur. Patients should be counseled for future drug avoidance. In our case, the patient was given supportive management along with adjunct systemic glucocorticoids. The patient did not develop complications and had a normal obstetric course.

Although rare terbinafine-induced SJS/TEN can occur. Thorough medication history, prompt discontinuation of offending agents and early intervention will lead to a favourable fetomaternal outcome.
